# Hypoxia-driven metastatic progression in synovial sarcoma: insights from SYO-1 and SW982 models

**DOI:** 10.1186/s12885-025-15125-5

**Published:** 2025-10-31

**Authors:** Maria Fueth, Jannis Christoffel, Kamran Harati, Felix Reinkemeier, Sonja Verena Schmidt, Marius Drysch, Flemming Puscz, Jannik Hinzmann, Tom Alexander Huyghebaert, Alexander Fiedler, Marcus Lehnhardt, Christoph Wallner, Yonca Steubing

**Affiliations:** 1 Department for Plastic and Hand Surgery, BG University Hospital Bergmannsheil Bochum, Ruhr University Bochum, Bürkle-de-la-Camp Platz 1, Bochum, 44789 Germany; 2 Department for Trauma and Orthopaedic Surgery, Cologne Merheim Medical Center, Witten/Herdecke University, Ostheimer Str. 200, Witten, Cologne, 51109 Germany

**Keywords:** Tumor microenvironment, Synovial sarcoma, Hypoxia, Metastasis, SS18-SSX fusion, Hypoxia-induced tumor progression

## Abstract

**Background:**

Synovial sarcoma (SS) is a rare but aggressive soft tissue malignancy characterized by a high rate of pulmonary metastasis and limited response to conventional therapies. Hypoxia, a common feature of tumor microenvironment, has been implicated in cancer progression, yet its specific contribution to metastatic dissemination in SS remains insufficiently characterized.

**Methods:**

We investigated the effects of hypoxia on metastatic behavior in two SS cell lines: SYO-1 (SS18-SSX2 fusion-positive) and SW982 (fusion-negative). Cells were cultured under hypoxic (O₂ < 1%) and normoxic (21% O₂) conditions, followed by reoxygenation. Expression of hypoxia-responsive and metastasis-related genes (e.g., HIF-1α, CA9, VEGF, IGF2, ADM, YB-1, TGF-β1) was assessed via qRT-PCR. To evaluate in vivo metastatic potential, a lung colonization model was established by injecting pretreated cells into the tail veins of immunodeficient (NMRI nu/nu) mice.

**Results:**

Hypoxia significantly upregulated canonical HIF-1α targets in both cell lines, with SYO-1 showing stronger and more sustained induction, particularly of CA9 and VEGF. In vivo, SYO-1 cells formed significantly more micrometastatic lung lesions compared to SW982, with distinct perivascular clustering and signs of early intravasation. SW982 cells exhibited limited, diffuse infiltration and lower hypoxia-induced gene activation. These differences suggest that the SS18-SSX fusion may synergize with hypoxia signaling to enhance metastatic potential. Notably, HIF-1α, CA9, and IGF2 expression correlated with metastatic capacity, while TGF-β1 expression declined under hypoxia, indicating a dynamic regulation of prometastatic pathways.

**Conclusion:**

Our findings demonstrate that hypoxia promotes SS metastasis through activation of HIF-1α and related pathways. Fusion-positive SS cells appear particularly responsive to hypoxic cues, suggesting that targeting hypoxia-induced signaling could be a promising strategy to inhibit metastasis in SS. These results provide mechanistic insight into SS progression and support the integration of hypoxia-targeted therapies into future treatment strategies.

## Introduction

Soft Tissue Sarcomas are a rare class of malignancies, accounting for approximately 1% of all cancers and synovial sarcomas (SS) make up 8–10% of all soft tissue sarcomas, compared to the much higher incidences for common epithelial cancers such as lung cancer (12.4%) or female breast cancer (11.6%) [1]. SS arises from pathological changes in mesenchymal tissue, the embryonic mesodermal origin of connective and skeletal tissues [2].

It can arise at any age, however, it most frequently affects adolescents and young adults between the age of 15 to 35, with a peak incidence in the third decade of life [3, 4]. From an anatomical perspective, the predominant location of soft tissue sarcomas is in the lower extremities, accounting for approximately 59.5% of cases. This is followed by the trunk (17.9%) and upper extremities (13.1%) [2, 5]. Consistently, SS have been observed to manifest in close proximity to tendon sheaths and joint capsules [2]. Despite its low overall incidence, SS is clinically significant due to its aggressive behavior and high propensity for metastasis. These characteristics underscore the need to better understand the factors that drive SS progression and metastatic potential.

Currently, the clinical treatment for SS primarily involves local surgical resection, often combined with adjuvant radiotherapy and chemotherapy [6]. The involvement of surrounding tissues frequently limits the extent of surgical intervention and due to its aggressive nature and notable chemoresistance, treatment strategies for SS have remained largely unchanged over the past two decades [3]. Even after radical resection, the risk of local recurrence persists unless adjuvant radiotherapy is applied which often only marginally improves long-term survival [7]. In palliative care, chemotherapy moderately prolongs survival but is often associated with a significant reduction in quality of life [7, 8]. Recently, promising advances in immunotherapy have emerged, particularly in T-cell receptor (TCR)-based therapies [9].

Despite multimodal therapy, the prognosis for SS remains poor. The 5-year survival rate is approximately 50% for patients with localized disease and drops to around 15% for those with metastatic tumors, with long-term survival at 10–30% after 10 years [9, 10]. Up to 40% of SS patients develop metastatic disease, with pulmonary metastases being the predominant cause of mortality [2]. Morphologically, SS are classified into three main subtypes: monophasic (59.6%), biphasic (26.9%), and poorly differentiated/undifferentiated (7%), each with variable clinical behaviors but shared molecular pathology [11].

### Molecular and hypoxic drivers of synovial sarcoma progression

SS are molecularly defined by the pathognomonic translocation t(X;18)(p11.2;q11.2), found in >95% of cases, resulting in fusion of the SS18 gene with SSX1, SSX2, or rarely SSX4 [2, 10, 11]. SS18-SSX1 is the most common variant (60%) and has been associated with enhanced invasiveness via Transforming Growth Factor-β1 (TGF-β1) signaling and promotion of cancer stem cell-like traits [10, 12].

In addition to cell-intrinsic oncogenic drivers, it is well established that the tumor microenvironment particularly hypoxia (low oxygen tension) plays a pivotal role in cancer progression [13]. As tumors outgrow their blood supply, regions of low oxygen develop, triggering Hypoxia inducible factor-1α (HIF-1α) stabilization [14]. HIF-1α translocates to the nucleus, dimerizes with Hypoxia inducible factor-1β (HIF-1β), and activates genes that promote adaptation and survival under hypoxia [15, 16]. Hallmark features of this response include enhanced glucose uptake, elevated lactate production, and suppressed mitochondrial respiration, collectively shifting the cell’s metabolism toward glycolysis, the so-called the “Warburg effect” [17]. This metabolic reprogramming induces glycolysis shift, angiogenesis (via Vascular Endothelial Growth Factor (VEGF)), and extracellular pH regulation (via Carbonic Anhydrase 9 (CA9)) [18–21]. Clinically, high expression of HIF-1α and CA9 correlates with advanced tumor grade and poorer survival in sarcoma patients [20, 21]. HIF-1α was shown to also enhance expression of collagen-modifying enzyme Procollagen-Lysine,2-Oxoglutarate 5-Dioxygenase 2 (PLOD2), facilitating a collagen structure conducive to tumor cell migration and lung colonization [13, 22]. Ablation of HIF-1α in murine sarcoma models significantly impaired pulmonary metastases without affecting primary tumor size, highlighting that HIF-1α specifically governs metastatic dissemination pathways [13].

Beyond its canonical targets, hypoxia intersects with other signaling axes relevant to metastasis. Y-box binding protein-1 (YB-1), a stress-responsive protein, enhances HIF-1α translation, forming a positive feedback loop that promotes invasion and motility [23, 24]. Likewise, TGF-β1, a major epithelial-mesenchymal transition (EMT) driver, can be upregulated and synergize with HIF-1α signaling to induce mesenchymal phenotypes [23, 25]. In SS, where SS18-SSX already elevates TGF-β1, this effect may be magnified under hypoxic stress [22, 26, 27].

Other hypoxia-responsive genes relevant to SS include Insulin-like growth factor-2 (IGF-2) and adrenomedullin (ADM) promoting survival and resistance to anoikis [28]. ADM, a vasodilatory peptide induced by hypoxia, supports tumor angiogenesis, growth, and may contribute to metastasis via autocrine and paracrine signaling [28, 29].

This study aimed to investigate how hypoxia influences the metastatic potential of SS cells by modulating gene expression and cellular behavior. Specifically, we examined the expression of hypoxia-responsive and metastasis-related genes under normoxic and hypoxic conditions. We also established an in vivo lung metastasis model to identify potential therapeutic targets for this aggressive tumor type. To capture molecular variability, two SS cell lines were used: SYO-1, which carries the characteristic SS18-SSX2 fusion, and SW982, which lacks this translocation and represents a more chemoresistant and poorly differentiated phenotype [30]. By comparing these models, we explored how hypoxia-driven molecular alterations, particularly involving HIF-1α, VEGF, CA9, ADM, YB-1, IGF2, and TGF-β1, interact with intrinsic oncogenic pathways. We integrated in vitro gene expression data with in vivo lung colonization assays in nude mice to evaluate the impact of hypoxic preconditioning on metastatic behavior. In the context of existing sarcoma research, these findings provide insight into the clinical potential of targeting hypoxia-related pathways in SS therapy.

## Methods

### Cell culture and hypoxic treatment

Human SS cell lines SW982 and SYO-1 (Cell Line Service, Eppelheim, Germany) were cultured in Dulbeccos Modified Eagle Medium (Thermo Fisher Scientific, Waltham, MA) supplemented with 10% Fetal bovine serum (Hyclone, Logan, USA) and 1% penicillin/streptomycin (ICN, Aurora, USA) under standard conditions (37 °C, 5% CO₂; Heraeus, Kendro, Langenselbold, Germany). Cell morphology was monitored daily via phase-contrast microscopy (Axiovert 100, Zeiss, Jena, Germany). For adaptation to serum-free culture, Panexin NTA (PAN-Biotech, Aidenbach, Germany) was gradually introduced. Cells were expanded until a final number of 4 × 10⁷ per cell line was reached.

On the day prior to the start of the experimental treatments, 2 × 10⁷ cells per condition (per oxygenation state) were seeded into culture vessels and incubated for 24 h under either hypoxic (O₂ < 1%) or normoxic (21% O₂) conditions at 37 °C and 5% CO₂ using a tri-gas incubator (NBS, Eppendorf, Hamburg, Germany). Following treatment 5 × 10⁶ cells per condition were harvested for Ribonucleic acid (RNA) and protein extraction.

### RNA isolation and quantitative RT-PCR

Following a 24-hour hypoxic incubation and subsequent reoxygenation phase, cells were harvested for total RNA extraction. Total RNA was extracted using the RNeasy Mini Kit (Qiagen N.V., Venlo, Netherlands) following the manufacturer’s protocol. The RNA samples (30 µL) were stored at −80 °C until reverse transcription into complementary Deoxyribonucleic acid (cDNA) was performed using quantitative real-time PCR.

To assess the efficacy of hypoxic treatment, the expression levels of established hypoxia-responsive genes were analyzed Quantitative real-time PCR (qRT-PCR) was subsequently performed using gene-specific primers. Target gene expression was normalized to 18 S rRNA and analyzed using the ΔΔCt method. Assessed genes were CA9, HIF-1α, VEGF, YB-1, IGF-2, TGF-α and Adrenomedullin.

### Protein isolation

Protein isolation was performed using the Pierce™ BCA (Bicinchoninic Acid) Protein Assay Kit (Thermo Fisher Scientific, Waltham, MA, USA) according to the manufacturer’s protocol. Absorbance was measured using a microplate reader (ELX808, Agilent BioTek, Thermo Fisher Scientific). Protein samples were stored at −80 °C for later analysis. After extracting the proteins, the cells that had previously been exposed to hypoxia were reoxygenated under normoxic conditions (21% O₂) for six hours to induce changes in gene expression that would persist. After reoxygenation, samples were collected again for RNA and protein analysis, as previously described. Remaining cells were washed, resuspended in calcium-free PBS at a concentration of 5 × 10⁵ cells per 50 µL, and loaded into insulin syringes for injection.

### Lung metastasis model in athymic mice

All animal experiments have been approved to be in accordance with local, federal, and European animal protection regulations by the State Agency for Nature, Environment and Consumer Protection of North Rhine-Westphalia (LANUV NRW, Register/Legislation number 84-02.04.04.2014.A236) under § 8 of the animal protection law paragraph 1. The study followed the principles outlined in the Guide for the Care and Use of Laboratory Animals. Twelve-week-old- male and female Immunodeficient nude mice (NMRI nu/nu, Charles River Laboratories) were used as the in vivo model. These mice lack a thymus and are thus unable to mount an effective T-cell response against implanted tumor cells.

The study included a total of 40 athymic mice (NMRI nu/nu, Charles River Laboratories), divided into four experimental groups: (1) SYO-1 following hypoxic treatment (2), SYO-1 without hypoxic preconditioning (3), SW982 following hypoxic treatment and (4) SW982 without hypoxic preconditioning. This results in a sample size of *n* = 20 per SS cell line

All mice underwent a one-week acclimatization period to minimize stress-related variables. They were housed in groups with free access to food and water. On the day of injection, pre-filled insulin syringes containing calcium-free PBS cell suspensions were transported on ice to the animal facility. Animals were anesthetized using inhalation anesthesia. Initially, mice were placed in an induction chamber pre-filled with 5 vol% isoflurane in 3–4 L/min oxygen until loss of reflexes was observed. To prevent corneal drying, an ophthalmic ointment was applied to both eyes immediately after induction. Subsequently, anesthesia was maintained via a face mask delivering a gas mixture of 0.4–0.5 L/min oxygen, 0.75–1 L/min nitrogen, and 1.5 vol% isoflurane, mice were positioned in a restraining device for tail vein injection. The injection site was disinfected, and 50 µL of the cell suspension was administered intravenously. After injection, anesthesia was gradually discontinued. Mice were monitored continuously until full recovery from anesthesia and then returned to their cages.

Following injection, mice were monitored daily over a 4-week period. Body weight was recorded and clinical behavior was assessed. Day 28 was pre-defined as the study endpoint for all experimental groups based on prior pilot studies using HT1080 xenograft models [31]. On the 28th day all mice were euthanized via cervical dislocation followed by organ dissection and lung harvesting under sterile conditions. One half of each organ was preserved in liquid nitrogen (N₂) for molecular analyses, the other half was fixed in 5% buffered formalin for histological processing.

### Histological evaluation

Formalin-fixed lungs were stored at −80 °C and later sectioned using a cryostat (Leica CM1950, Leica Biosystems, Wetzlar, Germany). Lung lobes were embedded in OCT (Tissue-Tek) and cut at 10 μm. For histological assessment, hematoxylin and eosin (H&E) staining was performed according to standard protocols (Papanicolaou’s Solution 1a, Merck KGaA, Darmstadt, Germany) for general histopathological evaluation and detection of metastatic foci.

Dual immunofluorescence staining against human vimentin and murine CD31 was used to distinguish human tumor cells from mouse vasculature and to assess metastatic progression. Furthermore Human Ki67 staining was applied to evaluate proliferation within metastatic lesions. Nuclear counterstaining was performed using DAPI (4′,6-diamidino-2-phenylindole).

### Immunofluorescence staining

Immunofluorescence staining was carried out on acetone-fixed cryosections using primary antibodies against human vimentin (1:50), mouse CD31 (1:250), and human Ki67. Blocking steps included avidin-biotin and 2% Bovine Serum Albumin (BSA) in Phosphate-buffered saline (PBS). Secondary antibodies included biotinylated anti-rat and anti-rabbit Immunoglobulin G (IgG), followed by Cy3-conjugated streptavidin (Thermo Fisher Scientific, Waltham, MA, USA) and nuclear counterstaining with DAPI. Coverslips were mounted with RotiMount-FluorCare (Carl Roth). Evaluation of stained lung sections was performed using an inverted fluorescence microscope with live-cell imaging capability (Olympus IX3 Series, Evident Europe, Hamburg, Germany).

### Statistical analysis

For all statistical evaluations, differences between the experimental groups and cell lines were analyzed using analysis of variance (ANOVA) and the two-tailed Student’s t-test. A p-value of less than 0.05 was considered statistically significant. Data analysis was performed using SPSS version 14.0 (SPSS Inc., Chicago, IL, USA) and Microsoft Excel (Microsoft Deutschland GmbH, Unterschleißheim, Germany).

## Results

### Cell culture

**Fig. 1:**
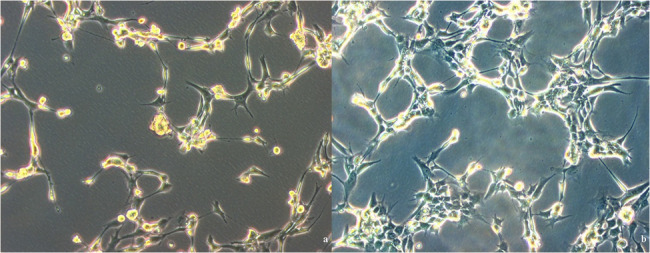
Native images of a cell culture flask (SYO-1) cultivated under Panexin NTA under normoxic (**a**) and hypoxic (**b**) conditions. The SYO-1 cell line was selected as an example (Scale 200 μm)

Under the specified culture conditions, both cell lines showed stable proliferation, with an average doubling time of 28 h for SYO-1 and 14 days for SW982. Cell viability and morphology were routinely assessed via phase-contrast microscopy. Representative images of SYO-1 cells under serum-free conditions are shown in Fig. 1a/b.

### Hypoxia alters gene expression in SS cell lines

Under normoxic conditions, cells appeared loosely distributed with distinct intercellular spacing. In contrast, hypoxic conditions led to markedly increased cell clustering and reduced spacing, while the overall morphology remained unchanged. These observations indicate a hypoxia-associated shift toward denser cellular aggregation.

### Hypoxia and reoxygenation alter gene expression in SW982 cells

**Fig. 2:**
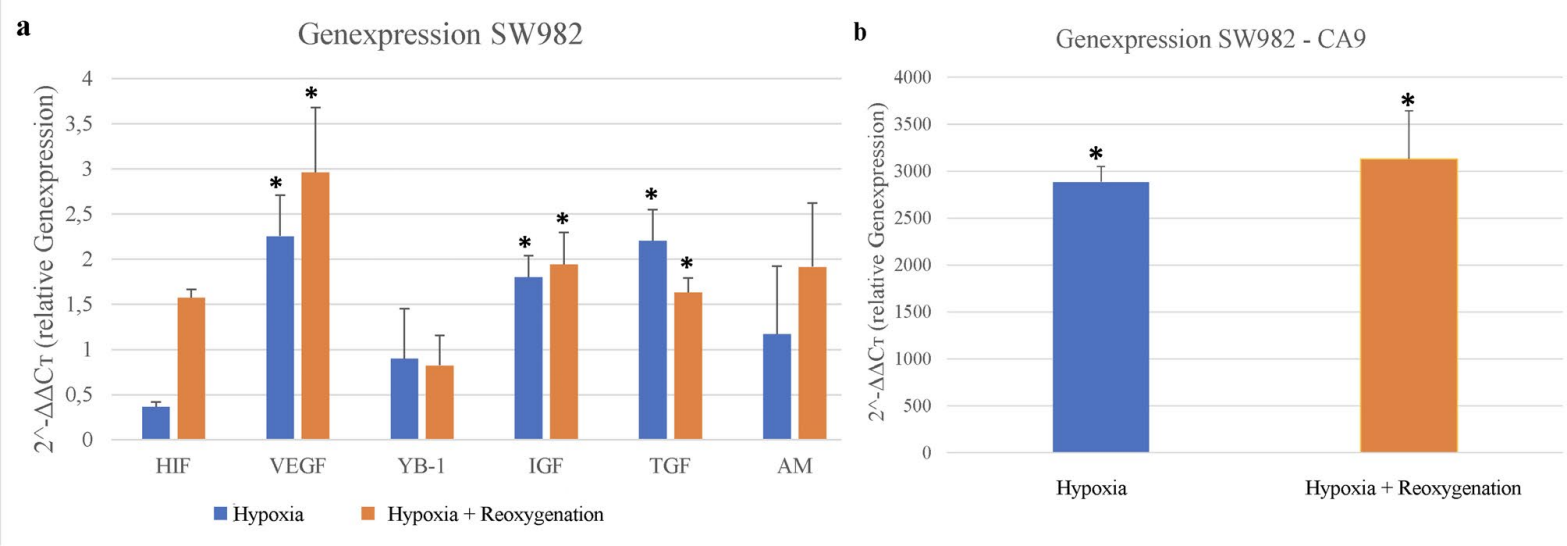
**a**/**b** Expression data of the SW982 cell line following hypoxia and hypoxia with subsequent reoxygenation. CA9 = Carbonic anhydrase 9, HIF = Hypoxia-inducible factor 1 alpha, VEGF = Vascular endothelial growth factor, YB-1 = Y-box binding protein 1, IGF = Insulin-like growth factor, TGF = Transforming growth factor, AM = Adrenomedullin. *p* value:* < 0.05, *** < 0.001

Figure 2a displays the relative expression levels of the analyzed hypoxia-associated genes. Due to its markedly elevated expression Carbonic anhydrase 9 (CA9) was plotted separately for better visualization of the remaining data (Fig. 2b). Most genes demonstrated an increase in expression after six hours of reoxygenation, with the exception of Y-box binding protein 1 (YB-1) and transforming growth factor alpha (TGF-α). A pronounced induction of HIF-1α expression was observed following reoxygenation **(**0.366 ± 0.05 vs. 1.57 ± 0.09**).** VEGF also showed a statistically significant increase **(**2.25 ± 0.45 vs. 2.97 ± 0.71, *p* = 0.0017). While adrenomedullin expression increased after reoxygenation **(**1.17 ± 0.76 vs. 1.92 ± 0.7**)**, this change did not reach statistical significance. IGF-2 exhibited a modest but statistically significant increase **(**1.80 ± 0.23 vs. 1.94 ± 0.35, *p* = 0.02), whereas TGF-α showed a significant decrease after reoxygenation **(**2.21 ± 0.34 vs. 1.63 ± 0.16, *p* = 0.001). For YB-1, no significant difference was observed **(**0.90 ± 0.56 vs. 0.82 ± 0.33**)**, and expression levels slightly declined post-reoxygenation. Due to its exceptionally high expression, CA9 was plotted separately (Fig. 2b). Relative expression of CA9 reached 2885.4 ± 165.11 under hypoxia and 3131.0 ± 517.31 after reoxygenation (*p* < 0.001). Thus, CA9 showed by far the highest induction among all investigated genes in the SW982 cell line.

### Hypoxia and reoxygenation alter gene expression in SYO-1 cells

**Fig. 3:**
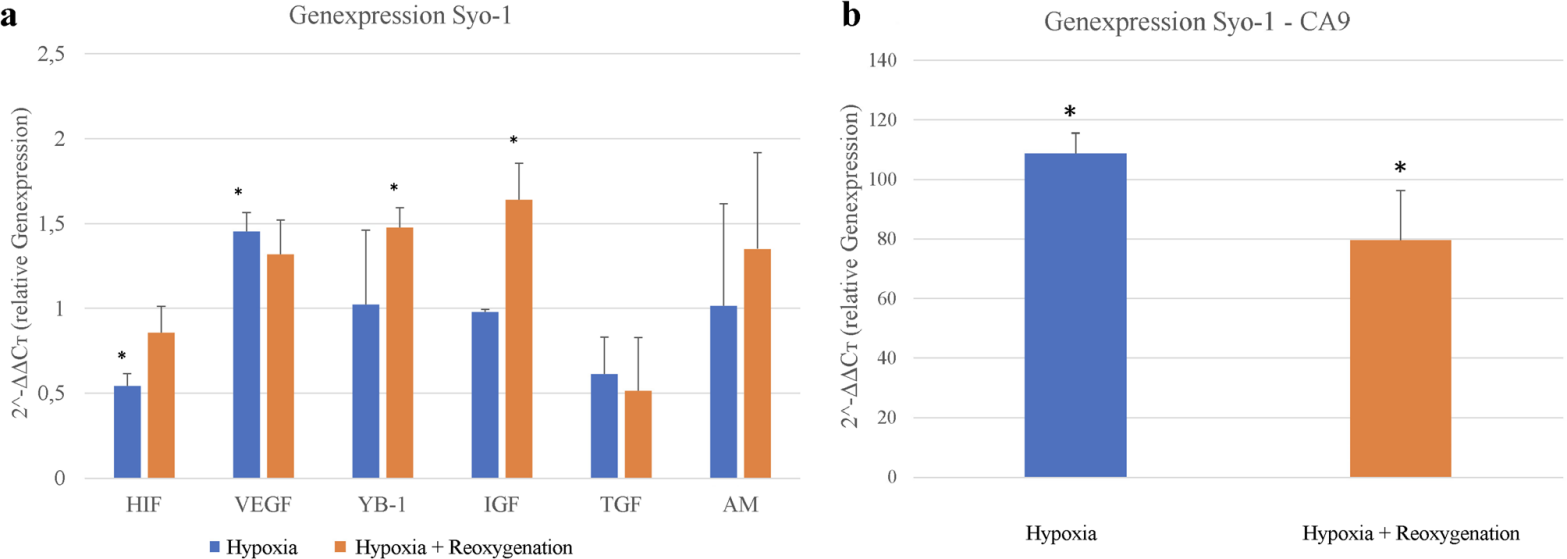
**a**/**b** Expression data of the SYO-1 cell line following hypoxia and subsequent reoxygenation. Values are presented as ΔΔCT and normalized to the housekeeping gene (18S rRNA) and normoxic control cells. CA9 = Carbonic anhydrase 9, HIF = Hypoxia-inducible factor 1 alpha, VEGF = Vascular endothelial growth factor, YB-1 = Y-box binding protein 1, IGF = Insulin-like growth factor, TGF = Transforming growth factor, AM = Adrenomedullin*. p* value: * < 0.05, *** < 0.001

Figure 3 illustrates the relative gene expression levels in the SYO-1 cell line. Due to the markedly elevated expression of the target gene CA9, a separate graph was generated. HIF-1α expression was moderately but statistically significantly increased under hypoxic conditions (0.54 ± 0.07) and rose incremental after reoxygenation (0.86 ± 0.15). VEGF expression also showed a statistically significant increase under hypoxia (1.45 ± 0.11), with a modest decrease following reoxygenation (1.32 ± 0.20). The YB-1 gene exhibited higher expression after reoxygenation (1.48 ± 0.12) compared to hypoxia alone (1.02 ± 0.44) (Fig. 3a). A similar trend was observed for IGF (1.64 ± 0.21 vs. 0.98 ± 0.02) and adrenomedullin (AM) (1.35 ± 0.57 vs. 1.01 ± 0.60), although these changes did not reach statistical significance (Fig. 3a). In contrast, TGF expression decreased following reoxygenation (0.51 ± 0.31) compared to hypoxia (0.61 ± 0.22), mirroring the pattern observed for VEGF. The highest level of gene expression was found for the hypoxia marker CA9, which reached 108.68 ± 6.88 under hypoxia and decreased significantly to 79.66 ± 16.80 after reoxygenation (*p* < 0.001), as shown separately in Fig. 3b.

### Histological evaluation of pulmonary micrometastases in murine SYO-1 and SW982 models following hypoxia treatment

**Fig. 4:**
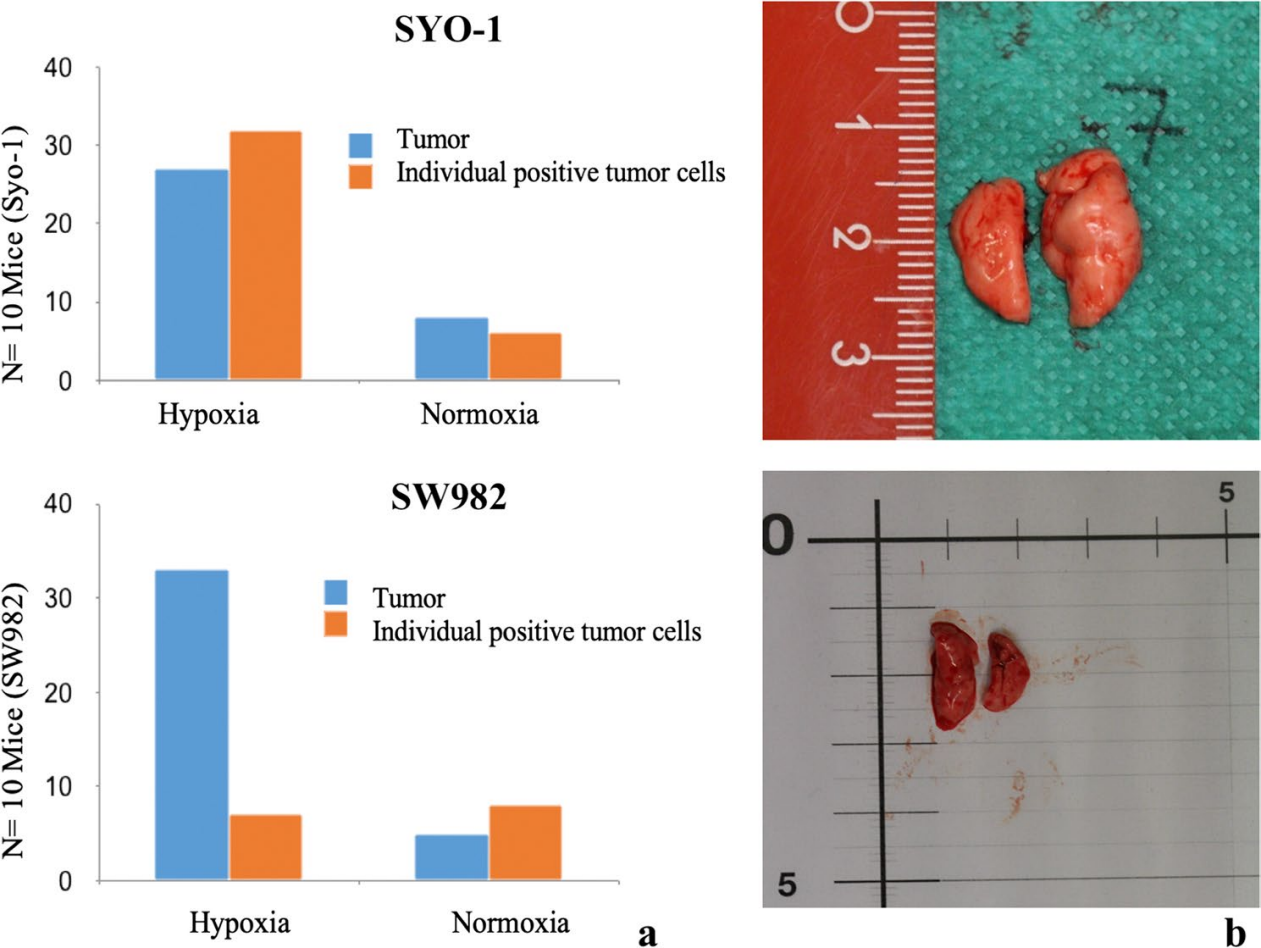
**a** summarizing the histological analysis of immunohistochemically stained lung sections 4b: Representative image of excised lung lobes from murine models.** b (**Top) Lungs from the SYO-1 cell line group, similarly prepared for downstream histological analysis. **b **(Bottom) Lungs from the SW982 cell line group, prepared for fixation in formalin or snap-freezing in liquid nitrogen

Figure 4a presents bar charts summarizing the histological analysis of immunohistochemically stained lung sections (*n* = 10 per cell line). Blue bars represent detected tumor clusters, while orange bars indicate individual positive tumor cells identified in the lung tissue of athymic mice. Each graph compares results from hypoxia-treated versus normoxia-treated groups. In the SYO-1 cell line, both the number of tumor clusters (27 vs. 8) and the number of positive single tumor cells (32 vs. 6) were markedly increased in the hypoxia-treated group compared to the normoxia group. In the SW982 cell line, a similar pattern was observed in tumor cluster counts (33 vs. 5); however, the number of individual positive signals was comparable between the hypoxia and normoxia groups (7 vs. 8). For the purpose of this analysis, any signal visibly comprising more than one cell was classified as a tumor cluster.

No clearly visible tumor manifestations could be identified during the macroscopic inspection of the lung specimens. The tissue showed no atypical changes, swelling, or nodular formations indicative of macroscopic metastases (Fig. 4b).

### Immunohistochemical analysis of SYO-1 and SW982 lung metastases 

**Fig. 5:**
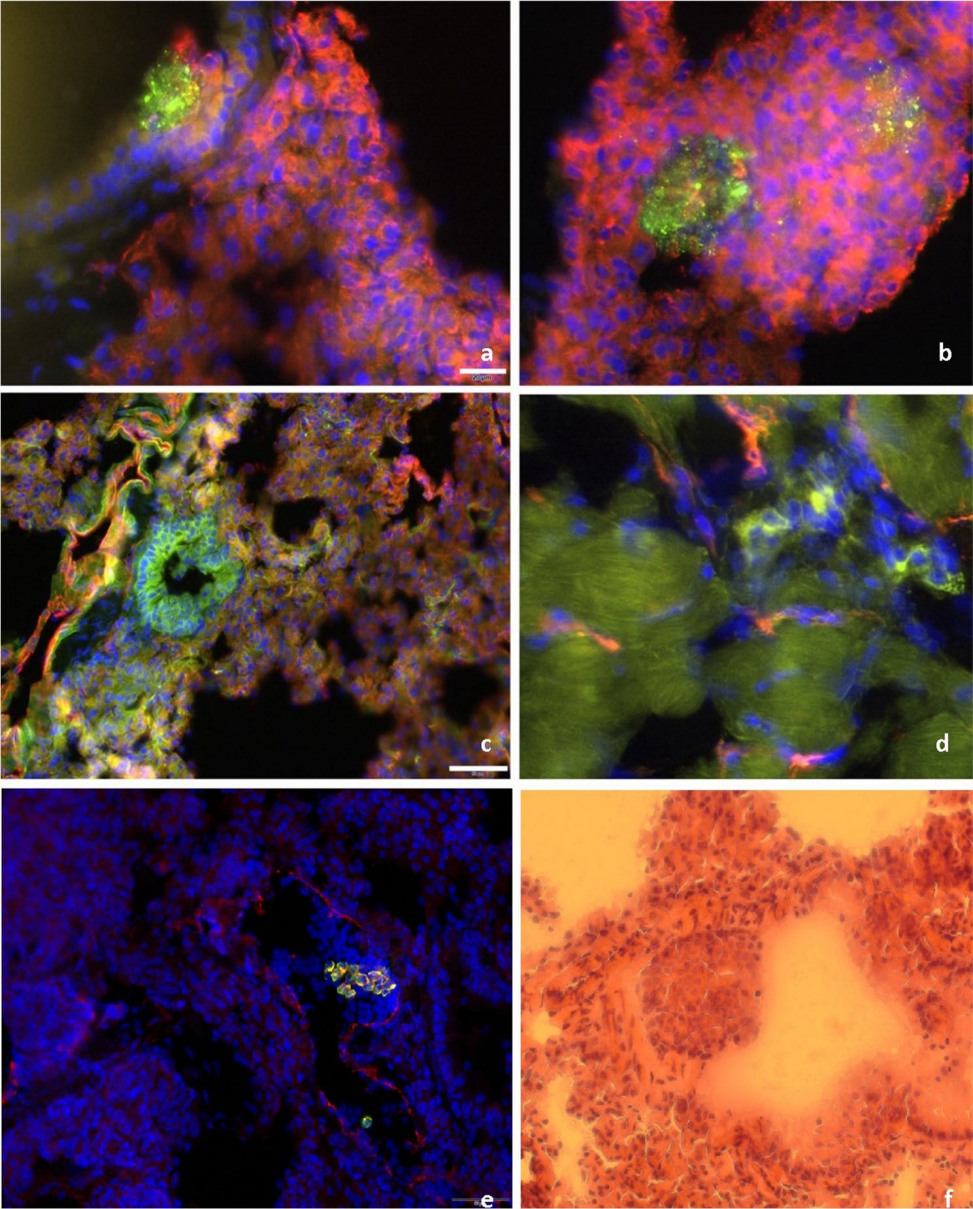
Immunohistochemical comparison of SYO-1 tumor cells (**a**-**b**) and SW982 tumor cells (**c**-**d**). Tumor cells are visualized using anti-human vimentin (green), endothelial structures with CD31 (red), and nuclear staining with DAPI (blue). **e** shows representative vimentin-positive SYO-1 tumor cells within lung tissue, with nuclei counterstained by DAPI. **f** presents an H&E-stained lung section from the same model, (scale bar: 50 μm)a/b/d: 20 μm; c/e/f: 50 μm

Figure 5 presents representative immunohistochemical images of SYO-1 and SW982 tumor cells in lung tissue. Tumor cells were stained with anti-human vimentin (green), vasculature with CD31 (red), and nuclei with DAPI (blue) (Fig. 5a-d). SYO-1 cells showed strong vimentin positivity and formed compact, nest-like clusters in close proximity to CD31-positive vessels, accompanied by dense nuclear accumulation. In contrast, SW982 cells appeared more diffusely distributed, forming larger aggregates that often extended into vascular lumina. High-resolution sections revealed individual vimentin-positive SW982 cells migrating into the surrounding lung parenchyma. Immunohistological analyses revealed distinct invasion patterns: SYO-1 cells preferentially localized in perivascular regions, forming compact clusters near endothelial structures, whereas SW982 cells exhibited a more diffuse distribution, infiltrating the lung parenchyma in a scattered manner.

Figure 5e displays a representative cluster of vimentin-positive SYO-1 tumor cells within a lumen-like structure bordered by CD31-positive endothelium, indicating intravascular localization and potential early intravasation. Partial yellow staining suggests initial extravasation. As observed previously, SYO-1 cells tend to localize perivascular without extensive parenchymal infiltration. Adjacent lung tissue shows dense nuclear architecture and preserved alveolar morphology, consistent with early-stage tumor spread. Supporting this, Fig. 5f presents an H&E-stained section revealing a compact tumor mass adjacent to a vessel, with intact surrounding alveolar structures and no evidence of deeper tissue invasion.

### Immunohistochemical detection of SW982 cells in lung tissue under hypoxia and normoxia

**Fig. 6:**
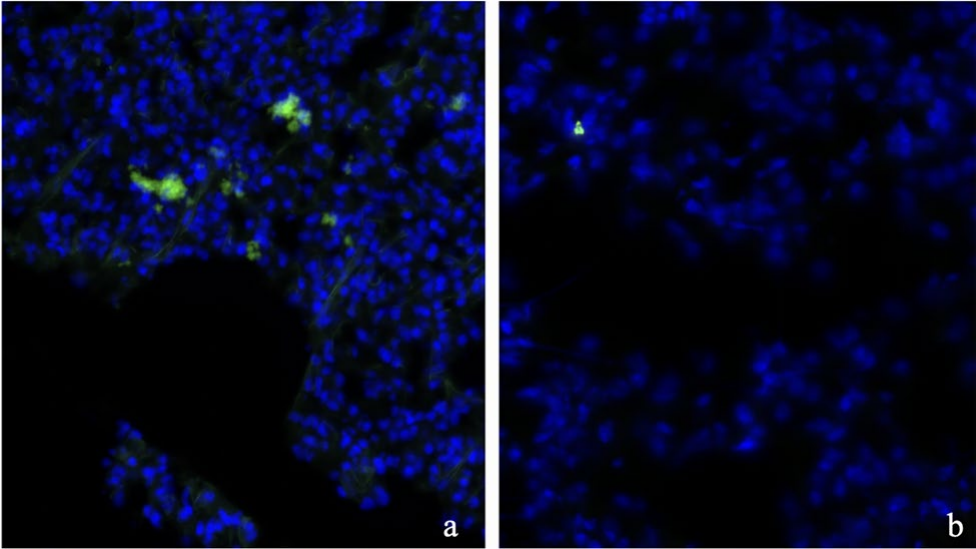
Representative detection of SW982 cells in lung tissue following hypoxia (**a**) and under normoxic conditions (**b**). Tumor cells are stained for human vimentin (green), and nuclei are counterstained with DAPI (blue). The scale bar indicates 50 µm in image a and 20 µm in image **b**

Figure 6 presents two immunohistochemically stained lung tissue sections from the SW982 cell line, with vimentin-positive tumor cells shown in green and nuclei counterstained with DAPI (blue). Unlike the intravascular localization seen in SYO-1 cells (Fig. 6), SW982 cells display a more diffuse distribution. Under hypoxic conditions (top panel), multiple tumor clusters are observed throughout the tissue without clear association to CD31-positive endothelium, suggesting extravascular localization and a more advanced invasive state. In contrast, under normoxic conditions (Fig. 6b), vimentin-positive cells are also present but appear less numerous and more sparsely distributed, indicating reduced infiltrative activity.

## Discussion

SS, although rare, pose a significant clinical challenge due to their aggressive behavior, chemo resistance, and high tendency for hematogenous metastasis, most commonly to the lungs. As per the current S3 guidelines for adult soft tissue sarcomas, treatment remains largely unchanged over the past decades and relies on surgical resection combined with radiotherapy and chemotherapy [8, 32]. However, therapeutic outcomes remain limited, particularly in metastatic cases, where prognosis remains poor and median survival seldom exceeds two years [3, 33, 34].

This study aimed to explore how hypoxia, a known hallmark of solid tumors, contributes to the metastatic potential of SS. Two cell lines were selected to reflect the biological heterogeneity of this entity: SYO-1 (SS18-SSX2 fusion-positive) and SW982 (fusion-negative). By combining in vitro hypoxia experiments with an in vivo metastasis model, we sought to identify gene expression changes and behavioral patterns relevant to early metastatic dissemination.

### Hypoxia triggers pro-metastatic gene expression changes

Hypoxia represents a central pathophysiological mechanism in the metastatic spread of sarcomas, particularly to the lung. The insufficient vascularization and rapid expansion of tumor tissue create hypoxic niches, which in turn activate HIF-1. Both cell lines, SW982 and SYO-1, responded to hypoxia by upregulating classical HIF-1 target genes such as VEGF, CA9, and ADM (Fig. 2/3). These changes align with the established role of HIF-1 in promoting angiogenesis, extracellular acid-base regulation, and resistance to cellular stress [13]. The induction of VEGF is consistent with the so-called “angiogenic switch”, enabling tumors to expand by promoting neovascularization [35]. At the same time CA9 was strongly upregulated in SW982 cells even after reoxygenation, suggesting long-lasting transcriptional effects of acid based regulation in maintaining cell viability in metabolically challenging tumor microenvironments [19]. It might represent a potential for adaptation to fluctuating oxygen conditions [20, 21]. These molecular changes translate into altered tumor behaviors. Elevated VEGF facilitates angiogenesis, thereby supporting tumor growth and providing entry routes for intravasation [18, 19, 35]. Increased CA9 expression has been linked not only to reduced apoptosis and enhanced survival but also to extracellular matrix remodeling, immune evasion, and therapy resistance [19, 25]. Together, these adaptations enhance the metastatic efficiency of sarcoma cells.

The clinical relevance of these findings is underscored by prior studies linking high expression of HIF-1α and CA9 to increased metastatic potential and poorer prognosis in soft tissue sarcomas [20]. Our data provide mechanistic support for this correlation: the hypoxia-induced upregulation of CA9 and other effectors likely enhances tumor cell survival under stress, facilitates extracellular matrix degradation, and supports early steps in the metastatic cascade.

### Differences in cellular behavior between fusion-positive and -negative cells

SYO-1 cells (SS18–SSX2 fusion-positive) consistently demonstrated a higher metastatic capacity in vivo, forming compact perivascular clusters and isolated micrometastases. In contrast, SW982 cells (fusion-negative) exhibited a more diffuse infiltration pattern and generated significantly fewer metastatic foci, which correlates with their comparatively weaker induction of hypoxia-responsive genes. The presence of the SS18-SSX2 fusion in SYO-1 cells likely contributes to this phenotype by enhancing pro-survival pathways, such as IGF2/IGF1R signaling, which are implicated in resistance to anoikis and increased metastatic competence [36, 37]. Interestingly, hypoxia-induced upregulation of IGF2 in SYO-1 cells may further support survival during detachment and circulation. Moreover, IGF signaling is known to activate the PI3K/Akt pathway, which can stabilize and enhance HIF-1α translation even under normoxic conditions [37]. This suggests that fusion-positive cells might maintain a pseudo-hypoxic state via autocrine IGF2-Akt signaling, a mechanism described in other tumor types and referred to as “growth factor-driven HIF activation” [38]. As a result, SYO-1 cells may possess baseline HIF-1 activity even in the absence of environmental hypoxia, which becomes further amplified under true hypoxic stress. This dual activation may explain their enhanced metastatic behavior [12]. Prior studies investigated that inhibition of this axis can induce apoptosis and reduce tumorigenesis [16, 37]. In contrast, SW982 lacking this autocrine IGF2 loop would rely solely on environmental hypoxia to activate HIF-1. This could partially explain why SW982 formed very few metastases. It may not achieve the same level of HIF-1 activation and metabolic adaptation as SYO-1, which might make SW982 cells more vulnerable when they attempt to metastasize.

### Hypoxia augments TGF-β1/EMT signaling

One novel observation of this study is that hypoxia led to a decrease in TGF-β1 mRNA, particularly in SW982 (Fig. 2a). TGF-β1 is typically considered a potent promoter of EMT, which facilitates cell migration and metastasis in various cancers [12, 27]. Its reduction in a hypoxic microenvironment may appear paradoxical but could suggest an early-phase adaptation in sarcoma progression [39]. This finding implies that hypoxia may temporarily suppress TGF-β1 signaling to prioritize alternative prometastatic pathways, such as HIF-1α-mediated angiogenesis or CA-9-associated acid-base regulation [19]. Alternatively, the decreased TGF-β1 expression might reflect lineage- or mutation-specific responses, particularly in the SS18-SSX-negative SW982 cells, which may utilize different mechanisms for hypoxia adaptation.

### YB-1 and hypoxia interplay

YB-1 mRNA was not significantly upregulated under acute hypoxia, yet YB-1 protein is known to enhance HIF-1α translation and amplify hypoxic responses. This highlights that gene expression alone does not capture the full regulatory picture, YB-1 may be activated post-transcriptionally through stress-related mechanisms such as phosphorylation or relocalization [24]. Literature reports that YB-1 is a crucial mediator of metastasis in sarcomas it promotes EMT by translationally upregulating factors like HIF-1α [23]. In our context, the presence of YB-1 in both lines likely reinforces HIF-1α activity during hypoxia, underscoring a regulatory network. Experimental studies have shown that YB-1 inhibition can reduce metastasis, making it a potential therapeutic target in hypoxia-driven progression in SS as well [23].

### In vivo metastasis outcomes correlate with in vitro hypoxic signatures

The dramatic difference in lung metastasis formation (SYO-1 >SW982) is in line with what one would predict from their gene expression. SYO-1 had higher angiogenic and survival factors (VEGF, ADM, IGF2) these likely facilitated establishing blood supply in metastatic sites and resisting apoptosis [28]. Additionally, SYO-1’s ability to form sizable metastatic nodules that then became hypoxic and showed HIF-1α positivity indicates an ongoing role of hypoxia in metastasis growth. Immunohistochemistry revealed strong HIF-1α expression in metastatic foci, suggesting that metastatic outgrowth maintains or reinduces a hypoxic program. This emphasises the concept of “hypoxia tolerance,” in which tumor cells leverage low oxygen conditions to adapt and thrive in distant tissues [40]. This is consistent with the general paradigm seen in other sarcomas: Eisinger-Mathason et al. demonstrated that deletion of HIF-1α in sarcoma models did not impede primary tumor growth but significantly suppressed metastatic progression. Mechanistically, this was linked to impaired expression of PLOD2 which is regulated by HIF-1α and critical for structuring the extracellular matrix in a way that facilitates tumor invasion [13].

Our SYO-1 cells presumably have intact HIF-1α and other invasion-related genes. The net result is successful metastasis. SW982’s failure to metastasize could be likened to a scenario of “incomplete hypoxic response” perhaps it cannot induce all necessary HIF targets or lacks YB-1/TGF-β boosts, thus it cannot perform the metastatic cascade effectively.

### Clinical implications and future directions

Our findings underscore the importance of hypoxia as a driver of early metastasis in SS. Tumors with hypoxic regions may harbor cell populations primed for dissemination via HIF-1α and YB-1-driven pathways [16, 23]. The hypoxic response could serve as a prognostic marker and therapeutic target for instance, via imaging with hypoxia tracers or by immunohistochemistry for CA9/HIF-1α in tumor biopsies. From a translational perspective, our data highlight CA9 as a promising membrane-associated therapeutic target [41]. The significantly increased expression of CA9 observed in both Syo-1 and SW982 cell lines by qPCR suggests its relevance across SS subtypes, reinforcing the rationale for preclinical testing of CA9-directed therapies. Moreover, pharmacologic strategies such as Hypoxia-activated prodrugs, HIF-1 inhibitors, or TGF-β blockers could be explored in combination with standard modalities and represent promising avenues for intervention in combination with conventional therapies [42].

Patients whose tumors show high HIF-1α or CA9 might have a higher risk of metastasis, as suggested by the correlation studies. While none are standard yet for sarcoma, our results support investigating such agents. For example, inhibiting HIF-1α activity might prevent the upregulation of genes like PLOD2, VEGF, CA9, and ADM, thereby stunting the processes needed for metastasis like invasion, angiogenesis and the survival in circulation.

### Limitations

While our study provides valuable insights, it has limitations that should be acknowledged. This study employed a tail vein injection model, which bypasses early metastatic steps like intravasation. Future studies using orthotopic tumor models would allow more comprehensive analysis of hypoxia in the primary tumor microenvironment. Moreover, broader transcriptomic or proteomic profiling could uncover additional hypoxia-regulated metastasis genes, including HIF-2α (EPAS1), PLOD2 or LOX, which were not assessed here but are known targets involved in matrix remodeling. Another important limitation of our study is that the contribution of the SS18-SSX fusion to hypoxia-driven metastasis was inferred from comparative behavior of SYO-1 and SW982, but not directly tested. Although our findings suggest that SS18-SSX-positive SYO-1 cells show enhanced responsiveness to hypoxic signaling, definitive evidence would require functional experiments. Although our design kept cells in normoxia until injection, in real tumors, cells experience hypoxia before entering the circulation. One might hypothesize that hypoxia-conditioned SYO-1 cells would be even more metastatic than normoxic SYO-1 cells, and perhaps hypoxia conditioning could enable SW982 to form some metastases where it previously could not. Testing this would simulate the clinical scenario more closely and could offer additional evidence of hypoxia’s role.

### Comparisons to other sarcoma subtypes

The role of hypoxia in promoting metastasis is not unique to SS. Similar mechanisms have been observed in osteosarcoma, Ewing sarcoma, and other soft tissue sarcomas, suggesting a universal pathway across multiple sarcoma subtypes [43]. These findings strengthen the rationale for integrating hypoxia-targeted strategies into sarcoma treatment. For example, recent data demonstrated that TH-302 (evofosfamide), a hypoxia-activated prodrug, exerts strong anticancer activity in Ewing sarcoma cells under low-oxygen conditions by inducing DNA damage, mitochondrial dysfunction, and apoptosis, independent of p53 status [44].

From a translational standpoint, the observed molecular and phenotypic adaptations suggest two complementary therapeutic approaches. Perivascular invasion patterns, as observed in Syo-1 cells, may be more effectively addressed with anti-angiogenic therapies. Conversely, the diffuse infiltration pattern of SW982 cells highlights the potential value of targeting migratory pathways or CA9-mediated pH regulation. Thus, hypoxia not only represents a driver of tumor progression but also a context-dependent therapeutic vulnerability.

## Conclusion

In conclusion, our study shows that hypoxia significantly contributes to the metastatic potential of SS cells by activating a cascade of molecular changes (HIF-1 targets, YB-1, TGF-β1, IGF2) that promote angiogenesis, invasion, and survival. The presence of the SS18-SSX fusion oncogene intensifies these effects, as seen by the highly metastatic behavior of SYO-1 cells relative to fusion-negative SW982. These findings highlight hypoxia as a critical microenvironmental factor in SS metastasis. Targeting hypoxia-driven pathways, alongside conventional treatments, could represent a novel therapeutic avenue to hinder metastasis and improve outcomes for patients with this aggressive disease.

## Data Availability

The datasets generated during and/or analysed during the current study are available from the corresponding author on reasonable request.

## Abbreviations

ADM: Adrenomedullin
ANOVA: Analysis of variance
BSA: Bovine Serum Albumin
CA9: Carbonic Anhydrase IX
cDNA: Complementary DNA
CT: Cycle Threshold
DAPI: 4′,6-diamidino-2-phenylindole
DNA: Deoxyribonucleic acid
EMT: Epithelial-mesenchymal transition
H&E: Hematoxylin and eosin
FBS: Fetal bovine serum
HIF-1α: Hypoxia inducible factor-1 α
HIF-1β: Hypoxia inducible factor-1 β
HLA: Human leukocyte antigen
IGF: Insulin-like growth factor
IgG: Immunoglobulin G
LANUV: Landesamt für Natur, Umwelt und Verbraucherschutz Nordrhein-Westfalen
N₂: Liquid nitrogen
PBS: Phosphate-buffered saline
PCR: Polymerase chain reaction
PLOD2: Procollagen-Lysine,2-Oxoglutarate 5-Dioxygenase 2
RNA: Ribonucleic acid
rRNA: ribosomal Ribonucleic acid
SS: Synovial Sarcoma
TCR: T-cell Receptor
TGF: Transforming Growth Factor
qRT-PCR: Quantitative real-time polymerase chain reaction
VEGF: Vascular Endothelial Growth Factor
YB-1: Y-box binding protein-1
